# Towards characterization of Female Genital Mutilation (FGM) in rural Nigeria

**DOI:** 10.4314/ahs.v20i4.55

**Published:** 2020-12

**Authors:** Amelia Ngozi Odo, Samuel Ifeanyi Christian Dibia, Evelyn Nwanebe Nwagu, MaryJoy Umoke, Prince Christian Ifeanachor Umoke

**Affiliations:** 1 Department of Human Kinetics and Health Education, University of Nigeria, Nsukka 410001, Nigeria; 2 Department of Public Health, Ebonyi State Ministry of Health, Abakaliki. Nigeria

**Keywords:** Female genital mutilation, qualitative study, practice, health implications, Nigeria

## Abstract

**Background:**

Female genital mutilation (FGM) is a public health challenge and seems to be secretly practiced in some rural communities, despite the ban in Nigeria.

**Objectives:**

The study aimed to identify the activities that are involved in FGM, type(s) of FGM practiced and the knowledge of health implications of FGM among rural community members in Ebonyi State, Nigeria.

**Methods:**

We employed exploratory design using qualitative technique. In-depth interviews were conducted with 44 adult (18 years and older) volunteers in four rural communities in Ebonyi State, Nigeria. After thematic analysis using NVivo 11 Pro software, eight sub-themes emerged, among which are: types of FGM practiced, seasons for FGM, FGM by health workers and community leaders, punishment for refusing FGM and knowledge of health implications of FGM.

**Results:**

Findings show that FGM is more like a process than just an act, and type most practiced in the study area is Type 1. Circumcisers are health workers and women leaders. Knowledge of health implications of FGM was found to be low among those interviewed.

**Conclusion:**

Based on the findings, we concluded that FGM is still practiced in some rural communities in Nigeria, maybe because of poor knowledge of health implications of FGM.

## Background

Female Genital Mutilation (FGM) is a public health challenge and needs to be given adequate attention. The World Health Organization-WHO 1 defined FGM as all the procedures which involve partial or total removal of the external female genitalia and/or injury to the female genital organs, either for cultural or any other non-therapeutic reasons. An estimate of more than 200 million girls and women around the world have undergone FGM in about 30 countries that practice it^2^. Though, most prevalent in African and Asian countries, FGM was also seen sparsely in UK 3 and United States 4 but is on the increase as more women migrate from African and Asian countries that practice FGM to US and UK^5^. African countries with highest prevalence of FGM (above 80%) according to UNICEF 6 include Egypt, Mali, Sudan, Somali, Guinea Bissau, and Sierra Leone, while Ethiopia, Mauritania, Liberia, and Burkina Faso had high prevalence between 51 and 80 per cent. Nigeria is one of the countries that recorded the prevalence between 26 and 50 per cent.

Many communities and ethnic groups in Nigeria subject the girl-child or women to the unhealthy cultural practice of FGM either openly or secretly. The prevalence of FGM in Nigeria was 41 per cent in 2001 7 and 27 per cent in 2013, with variations across the regions and the highest in the southern part of the country 6. The Nigerian 2013 National Demographic and Health Survey-NDHS 8 however, reported that 25 percent (a little lower than UNICEF report) of Nigerian women are circumcised, with Ebonyi State having the highest percentage (74.2%) the southeastern Nigeria. The actual prevalence may be higher than the recorded figure because of the elimination campaign which seems to make the practice more secretive. However, with improved education and social status of women, many women who underwent FGM disapprove of the practice while some still subject their daughters to FGM just to protect them from being ostracized, shunned or disgraced 9. The continued practice of FGM has been attributed to cultural beliefs and values of the individual communities and ethnic groups 10.

Female genital mutilation may involve partial or total removal of the female external genitalia. There are four types of FGM. Type I, II, III and IV. Type I, II and III are the most practiced in Nigeria 1, 11. Type I (clitoridectomy) involves removal of the clitoral hood (prepuce) with or without removal of all or parts of the clitoris. Type II (excision) involves removal of the clitoris together with part or all the labia minora. Type III (infibulation) involves removal of part or all the external genitalia (clitoris, labia majora and minora) and stitching or narrowing of the vaginal opening, leaving a very small opening to allow for the flow of menstruation. Type IV which is rarely practiced involves the introduction of corrosive substance into the vagina 11. The type practiced depends on region and different cultural settings 12. The Southeastern region of Nigeria where the present study was carried out, practice type II FGM mostly 13.

The type of FGM practiced by any group notwithstanding, FGM is physically, socially and mentally unhealthy and harmful. There are both short -and longterm consequences of FGM 14. Some of which include bleeding which could lead to anaemia, shock and death; severe pain which can also lead to shock; infection resulting from poor management of wound and use of unsterilized instruments, HIV, genital tissue swelling. Other consequences include prolonged labour, which may result to still birth and death of the mother, urinary incontinence, painful sexual intercourse 15 and lack of sexual desire which is the major reason for cutting off the clitoris 13. Other reasons for the practice of FGM in Nigeria have been recorded to include cultural purification, family honour, aesthetic reasons, and protection of virginity, giving a sense of beloging to a group, and increased sexual pleasure of husband 16. These reasons and the type of FGM practiced could differ based on region and socio-cultural variations. In Ebonyi State and other states in the southeastern Nigeria (where FGM is practiced), FGM is believed to reduce woman's sexual arousal and prevents extramarital sexual behavior 17.

Naturally, every mature male or female should have sexual feelings or desire. This is often aroused by touching some erogenous zones which the clitoris is one. The act of cutting off a woman's genitalia should not only be seen as harmful but also dehumanizing 15, infringement on the human and reproductive rights of the individual^18^. Poor understanding of these rights and knowledge of harmful effects of FGM seem to have allowed the values posed on the practice culturally to grow deep. This could be explained by the theory of reasoned action.

The theory of reasoned action (TRA) was propounded by Fishbren and Ajzen in 1967. The theory assumes that most behaviours of social relevance are under the willful control. It asserts that the most important direct determinant of behaviour is behavioural intention^19^. The theory explains that a person's intention to perform or not to perform the behaviour is the immediate determinant of that behaviour, and that a behavioural intention is determined by a person's attitude to the behaviour which is determined by beliefs about the outcome of the behaviour and evaluation of the expected outcome and subjective norms. In the study area, it has been observed that some rural communities still value FGM and accords more respect to the circumcised females than uncircumcised. So, most people engage in the practice to satisfy the community norm or respected ones. Could it be that they are not aware of the negative outcome or health implications of FGM? Or that there are other “non-harmful” forms of FGM practiced in the rural communities? This study, therefore, sought to explore the practice of FGM and knowledge of health implications of FGM among adults (18 years and older). We believe that this group of people could give us more information as they must have discussed or practiced FGM.

## Methods

### Study area

The study was conducted in Ebonyi State, Nigeria. Ebonyi State is one of the 36 states of Nigeria, situated in the South-eastern zone of the country. The state is bounded by four states in Nigeria: in the north by Benue State, in the West by Enugu State, in the East by Cross-River State, and in the South by Abia State. The prevalence of FGM in Ebonyi State in the last Nigeria Demographic and Health Survey-NDHS was 74.2% 8. The population of Ebonyi State during the 2006 national census was 2,176,947 with a projection of 2,880,400 in 2017 and land mass of about 5,670 km^2^ 20. Majority of the population live in rural communities with similar cultural heritage. Four of these rural communities were purposively selected and used for the study.

The communities are: Inikiri Effium and Akparata Effium both in Ohaukwu LGA, Odeligbo in Ikwo LGA and Ofiaorji in Abakaliki LGA. These communities were chosen due to their rural and cultural background that makes the FGM practice more valued. Some of the communities are hard to reach areas because of their distance and lack of good roads. They also lack good health facilities. Each of the communities only has one health centre serving about 10,000 to 12,000 populations. Most of the community members are predominantly farmers and home delivery is very common. There are more traditional birth attendants and unqualified health personnel in the communities. Factual health information and campaign against FGM is rarely provided until recently by the State governor's wife and the Amanda Marga Universal Relief Team (AMURT)-Nigeria. The AMURT, which is a non-governmental organization, has extended their activities aimed at reducing maternal and neonatal mortality to include campaign against FGM in all the rural communities in the state. Despite all of these, FGM is still practiced in these rural communities.

### Design, sample selection and inclusion criteria

The exploratory research design was used to explore the practice and knowledge of health implications of FGM among the adult rural community members in Ebonyi State, Nigeria. In-depth interviews were conducted among adult males and females that were recruited through a non-governmental organization (AMURT) in the state that has been working in the rural communities. Amanda Marga Universal Relief Team (AMURT) Nigeria is a non-governmental organization with the aim of reducing maternal and neonatal mortality in the Ebonyi State 21. Four AMURT staff were trained and used to recruit adults that were interviewed from each community. In the study area, FGM is becoming a secret practice, so, people do not freely discuss it. This made us to employ two sampling techniques: convenience and voluntary sampling techniques. Convenience allowed the researchers to select people that were easy to reach, while voluntary was lastly used to include those that indicated willingness to give information, about FGM, after explanation of the study objectives. These techniques were employed to select 11 adults from each community, making a total of 44 participants (4 males and 40 females). Our initial target was to recruit and interview equal male and female participants in order to compare their responses, but it was very difficult to get the males to consent to talk about issues of FGM practice in all the four communities. We were able to get only four men from two communities. All adults from 18 years and older, male or female, circumcised or not and have lived up to 10 years in the study area, were eligible to participate in the study.

### Data collection and analysis

Initially, we liaised with the AMURT and agreed on getting assistance from them during participant sampling and data collection. Participants were invited by the AMURT to the nearest health centers where the interviews were conducted and helped during the interview to interpret the questions and responses in their local dialect. Participation was on voluntary basis. The participants assented to the interview orally and by signing the consent forms. Confidentiality of their identity (including gender) were assured and each person was encouraged to freely give every information he or she had about the FGM. We conducted 44 interviews within a period of three months (October to December 2017). We had only four males (Table 1) who agreed to speak on the issue of FGM in their community. We decided to include males in the study because they are law and decision makers in the communities and also to gain different gender perspectives on the practice of FGM. The male participants were more reserved with information than our female participants, most of whom gave detailed information they have about FGM in their communities. However, we reached data saturation at the point we had 44 interviews, when additional interviews yielded no new themes 22, and that informed our stopping at 44 interviews.

**Table 1:** Demographic characteristics of participants (n=44)

S/N	Characteristics	f(%)
1	**Age**	
	Mean	28.05
	Mode	32
2	**Gender**	
	Male	4(9.09)
	Female	40(90.91)
3	**Level of Education**	
	Primary	20(45.45)
	Secondary	15(34.09)
	Post-Secondary	6(13.64)
	No Formal	3(6.82)
4	**Marital Status**	
	Married	42(95.45)
	Single	2(4.55)
	Divorced/Separated/Widowed	0(0)
5	**Occupation**	
	Trading/Business	23(52.27)
	Civil servants	6(13.64)
	Artisan or Farming	9(20.45)
	Students/Unemployed	6(13.64)
6	**Religious Affiliation**	
	Christianity	40(90.91)
	Traditional	4(9.09)
	Islamic	0(0)
7	**FGM Status (Females only (n=40))**	
	FGM Survivors	9
	Non-victims of FGM	14
	Unwilling to disclose	17

The interviews were audio recorded and notes were also taken. Each interview had an interviewer, an interpreter and a recorder took notes of the responses. After the data collection, the audio recordings were translated and transcribed verbatim into English language using the express scribe transcription software. The notes were also integrated into the transcript taking cognizance of non-verbal cues of the participants. The transcripts were imported into NVivo 11 Pro software used to analyze the data. Two persons did the coding independently. To ensure quality of the data, the transcripts were reviewed after each transcription by the interviewers and transcribers, which helped the interviewers to improve their probing skills on the subsequent interviews. The codes of the two coders were compared and discussed^23^, after which/span>emerging eight themes (types of FGM commonly practiced, seasons for FGM, FGM performed by community elders and health workers, instruments for FGM, care of FGM, rewards for FG, consequences of FGM, and knowledge of health implications of FGM) were confirmed. The Framework approach 24 was employed for the data analysis. The researchers read the transcripts for familiarization and developed thematic frameworks. The transcripts were then coded under the thematic framework using NVivo 11 Pro software. Data under each theme were summarized and interpreted. Our University's ethical committee approved the study.

## Results

Table 1 shows that the average age of the participants is 28.05. Out of the 44 participants, 40 are females, 42 are married and 20 had only primary education. Majority (23) are traders or engaged in a business and 40 are Christians. Seventeen of the female participants chose not to disclose their FGM status, 14 were non-victims while nine are FGM survivors

### Types of FGM commonly practiced

The majority of participants in this study did not consider FGM in terms of the medical assessment (Type I, II, III, IV), but rather conceptualised circumcision as “*ugu umu sukul*” (child circumcision and “ugu umu okenye” (adult circumcision), both of which were practiced; “We do practice adult and child circumcision. They refer to child circumcision as “*ugu umu sukul*” (P3). Women are required to undergo both these procedures, and this indicates that circumcision is not a single event, but rather a set of cultural practices which the woman must engage with throughout her life. Ugu umu sukul is practice as Type 1 Clitoridectomy, and participants felt that the most common form in Ebonyi State. This is done at an early age (typically below 10 years of age), “*the one done at tender age for girls*” (P16). This is done when the girl child is still in primary school or below 10 years, and can be done at any time of the year. It requires specialized instruments and care like care of the wound and relief of pain. Ugu umu okenye is carried out when the woman is about to get married (no specific age range). This “*is being done by shaving the person. Some are done by applying cam wood powder (ushee in Igbo language) all over the person's head and private part*” (P1). Participants saw this as a continuation of the circumcision process; “*After child circumcision, you will undergo adult circumcision but they will not cut anything from the body of the woman only shave the private part of the woman*” (P4). However, others such as P12 indicated that this “*is just a traditional rite*”, and there was a possibility of the woman having choice, “*those that don't want will go straight to traditional marriage*” (P12).

### Seasons for FGM

Most participants were of the view that while child female circumcision can be done any time during infancy, adult female circumcision is usually done during the ‘*Aju*’ festival or the woman is about to marry. For instance, P8 stated “*During infancy female circumcision can be done any time. There is no special time or season for it. But for female circumcision done when the woman is grown, it is usually done during ‘Aju’ ceremony. This is usually around April*” (P8). Further to this, P12 added that “*It must be the period when planted yams must have matured, so that they might pound some of the yafor the person. They would take good care of the girl*”

### FGM performed by the community elders and health workers

FGM is mostly performed by older women in the community and also by health workers in the health facilities. “*It is usually done by the midwives (the people usually refer to all female health workers who attend to pregnant and nursing mothers including TBAs and patent medicine dealers as midwives)*” (P24). P6 explained that “*It used to be women who inherited the skill from their fore parents who conduct FGM but now because of orthodox medicine, they (those seeking for FGM) go to the hospital to get FGM and then they come back home to perform the traditional rituals associated with it*”. Another participant said “*The elders carry out the traditional FGM (the one carried on married women before they give birth) but the infant circumcision is done by midwives*” (P8).

To buttress the above point, P7 stated “*….in fact anybody who can deliver a woman of a baby was able to carry out female circumcision. In those days also, FGM were being carried out even in the hospitals but these days they no longer do it in the hospital*s” (P7).

### Instruments for FGM

The instruments mostly used for FGM currently were razor blade and scissors. There were other locally made sharp objects used but they are getting obsolete in the practice. Most of the interviewee reported using razor “*They use razor blade to mutilate some, but for me I was mutilated with scissors*” (P4). “*We use aguba (locally made sharp knife)” (P3). Scissors and the local blade are reused for several people until it gets blunt, without sterilization. “They just wash and keep for another time*” (P33).

### Care of FGM wound

After cutting off the clitoris, bleeding is controlled by pressure and the wound is massaged with hot water daily until the wound is healed. The person may be given drugs to relief her of pain. “*They will massage the person with hot water and give the person some pain-relieving medicine*” (P1). “*……after that they will use cotton wool or cloth to clean the blood and also apply hot water on it day and night*” (P5). However, some participants said that the wound care depends on where the FGM was performed and who did it. “*It depends on where the FGM was done. If it is done in the hospital, they will treat her, but if it is at home, they will call the chemist people (patent medicine dealers) to come and treat her at home*” (P13).

### Rewards for performing FGM

The communities promote FGM through cultural ceremonies and gifts to the girl during the adult circumcision. “*They promote this act by carrying the woman round the community, praising her as well. The woman is seen as an honoured and a complete woman*” (OP13). “*…..there were goats slaughtered for the purpose and given the women gifts just equivalent to those of wedding gifts*” (OP13). Moreover, the FGM survivor is placed on special diet for a period of three months and specially attended to. “*The woman circumcised in the traditional way will be well fed during the period of circumcision. Special meals will be prepared for her*” (P11).

“*The mother or the husband of the circumcised woman will buy fruit and other things to help the woman regain lost nutrient during the act for at least four months. The person will be fresh and plump during the time of the traditional celebration. On that day, the family members will carry different kinds of food on their head to show the people present what she eats when she was newly circumcised that made her look fresh and plump*” (P2).

Furthermore, “*after three months the husband of the woman will kill at least two goats for her and prepare a delicious soup for her like ogbono soup or egusi soup or meal that is usually prepared for women that put to bed for her to regain herself. The circumcised woman will look fresh, plump (physically healthy) and fair in complexion. She will stay in door until the day she will come out*” (P7).

### Consequences for refusing FGM

Refusal to perform FGM attracts punishments directly or indirectly. The person could be excommunicated or mocked by community members. She may also be maltreated by her husband and his family until she accepts to be circumcised. In the words of some participants, “*in the olden days, if a woman said that she will not be circumcised, the kindred will excommunicate the defaulters' family from them but it has started to change*” (P1). “*Well if the person refuses, her people may be parents or elderly members of the family will come and force the lady to do it*” (P11), and any “*woman who is not circumcised is regarded as a kid no matter her age*” (P22).

“*…. we see FGM as tradition so if a woman says that she is a Christian and therefore that she will not be circumcised, the husband will not be happy. Again, if a woman delivers a baby without been circumcised, she will be made fun of and accused of delivery with “akpapi” (akpapi is the clitoris which was believed to be ritually unclean). A woman who is circumcised before delivery is seen as fully matured for pregnancy and delivery but any woman who becomes pregnant without circumcision is seen as being pregnant with “akpapi' (Clitoris)*” (P7).

The uncircumcised were not allowed to attend cultural activities, events or ceremonies in the community. “*There are certain events you cannot attend because you were not circumcised. You don't even go to where they are performing circumcision and not to talk of touching anything there*” (P19). “*They do not allow such a person to come in the mix of elders. Even young people who are circumcised get share of anything in the community first before the older uncircumcised*” (P9). A participant noted that sometimes the females were forcefully circumcised. In her words, this participant said “*…like in the olden days, student's own (child circumcision) is compulsory for all females and if the person refuses to do it, her hands and legs will be tied with rope, while they perform it*” (P16).

### Knowledge of health implications of FGM

Majority of the participants knew only of the immediate physical health consequences of FGM like bleeding and pain. One of the circumcised participants said, “*it was painful, and I bleed at the same time*” (OP4). “*It is painful and causes bleeding*” (P25). “*I know that some people experience pain while some do not feel the pain. Some also bleed profusely but we see such people as having too much blood*” (P17). Furthermore, “*It causes bleeding during child delivery*” (P16). “*They said during childbirth that the females find it difficult in delivery*” (P15). “*I have a sister who was circumcised when she was young, the circumcision lead to constriction of her birth canal and she had to undergo surgery to have it corrected*” (P3).

Few of the participants knew the social health implications of FGM. Their knowledge was from the campaign against FGM by the State government. The office of the Wife of the Governor has a campaign programme aimed at ending FGM in Ebonyi State. This programme which was started in 2016 has helped in reducing the prevalence of FGM in State. A participant said “*…from teachings on FGM, we are told that a circumcised female does not enjoy sex with her husband and that sometimes it results to problems during delivery*” (P5).

## Discussion

Our study revealed that the type of FGM mostly practiced in Ebonyi State is Type I (Clitoridectomy), just as in many other countries in Africa, Middle East and Asia^25^, despite the provision of the Violence Against Persons (Prohibition) Act 2015 26, prohibiting the practice of FGM in Nigeria and the Law Abolishing Harmful Traditional Practices Against Women and Children in Ebonyi State 27. Type I FGM involves the removal of the clitoris 1, which is one of the sensitive parts of the female external genitalia that trigger sexual excitement^28^. The clitoris is cut off so that the woman will not have sexual desire and will not be easily aroused sexually, thereby increasing their ability to remain a virgin before marriage and faithful to the husband after marriage^2^. This act is against the sexual and reproductive rights of women, which are part of their human rights 29, 30. Every individual, male or female, is entitled to his or her sexual rights 31, and therefore should enjoy sex as much as the partner does. Moreover, we found out that FGM is done in two stages. The first being the cutting of the clitoris which is done during infancy, childhood, adolescence or any other period before the woman gives birth to a child. The second stage, which the communities referred to as “adult circumcision”, is the ceremonial aspect of FGM, done within the period the woman is getting married; before she is finally taken to her husband's house. There is no specific age range.

During this ceremony, the woman is taken to a hidden place and cross checked to ensure that the clitoris had been cut, after which she will be shaved and decorated with local powder. However, if during the check, the woman is found uncircumcised, she will be circumcised before continuing the ceremony. The woman stays indoor (fattening) during which she is feed with special meals and gifts are brought to the woman by family members and friends. This stage is generally accepted by many community members and could make some women to accept FGM. This stage of FGM is usually organized by every community in the state, at least once in a year.

There is culturally a season in a year during which “adult circumcision” is done in every community, but the “child circumcision” (cutting off the clitoris) has no season. Most communities perform the “adult circumcision” during a cultural ceremony called “Aju”, which is usually celebrated around April every year. However, some communities perform the ceremony when yams are being harvested. This is to ensure that the families of the circumcised have enough yams to prepare special pounded yam meals for the circumcised. The season is anticipated and warmly welcomed by community members because of the foods, drinks and dances. Again, the circumcisers looked forward to this period of time because they make more money. It is a source of income to the traditional circumcisers.

Traditionally, FGM is mostly performed by older women in the community 32 or the traditional birth attendants (TBAs), but surprisingly, we found out that some patent medicine dealers and health workers were performing FGM in their drug stores and health facilities, respectively. The study revealed that people used to go to the health facilities for the stage one, which is cutting off the clitoris, while the second stage, which is the initiation ceremony is done in the community by elderly women. Moreover, a baby girl can be circumcised by the person that conducted the delivery. Previous studies reported that FGM is solely done by community women leaders and TBAs 33, 34. The instruments used by any circumciser could depend on the place of circumcision and the circumciser.

Locally made sharp blade (aguba) was traditionally used for FGM. Currently, its use is becoming obsolete and the most widely used instruments are razor blade and scissors 35. The main problem is that we found out that most times one instrument is reused for different persons until it gets blunt, without proper sterilization. The much that could be done was to wash of the blood stain. This could be a source of transmitting infections from one person to another. Studies had shown that infectious diseases are transmitted through sharing of contaminated sharp objects 36. A well-known circumciser can circumcise more than five person a day, and can use one instrument for all, as such, both the instrument and wound may not be properly attended to.

Our study found out that the circumcisers have developed some skills to stop bleeding and care for the wound. They control bleeding by applying pressure to the area and subsequently use hot water to massage the wound until it heals. Pressure to a bleeding site helps constrict the blood vessels and bleeding will gradually stop 37. However, this may not work in some individuals with blood clothing factor problems 38. The bleeding may be uncontrollable, resulting to shock which can lead to death 39. Again, massaging with hot water alone may not protect the wound from being infected, instead, it could even increase the chances of wound infection, depending on the sterility of the materials used to massage. Pain relieving drugs are also given to the circumcised to help relief her of much pain that accompany circumcision. Some young girls bear the pain because of the gifts she will get after the final ceremony. There are many material benefits of FGM in the form of praises and rewards for accepting the act and for becoming fully inducted into the culture of the land. We learnt that during the ceremony, the girl is carried round the community, with songs of praise and honour. Furthermore, gifts are brought to her and fat goats killed and used to prepare special meals for her. Previous researchers had reported that reward enhances practice 40, therefore, these gifts, special meals and praises could be a strategy by the communities to keep promoting the practice of FGM in the State.

The sense of fulfillment and being accepted as a grown woman in her community may even make her feel socially and mentally healthy, as opposed to rejection, if she had refused to be circumcised.

Refusal to be circumcised in the rural communities attracts forms of consequences or punishments either directly or indirectly. Community, clans and family administer punishments to defaulters to uphold values, cultures and tradition 41. We learnt that previously, the punishment for refusing FGM could be as serious as excommunication of the entire family, but Christianity and the campaign by the government to stop FGM has helped in modification of the outright punishments. However, the woman will be mocked and may not be allowed to be in the mist of elders. Again she would not be allowed to attend cultural activities. These punishments could negatively affect the social and mental health of the woman 42, 43. Punishmnts could make a girl to accept FGM, which she would not accept to do ordinarily, resulting in anxiety disorders and other several personality disorders 44. The person can only accept the punishment when he or she is at abreast with the consequences of accepting the practice.

We found out that majority of our participants know of the immediate health consequences of FGM such as pain and bleeding 36. Even with this knowledge, some did not see them as serious reasons to stop FGM rather they referred to the victims of profuse bleeding as “those who have enough blood”. Profuse bleeding could result from cutting off of the clitoris or/and the labia, which may lead to anaemia and consequently to shock and death may occur 36. Previous studies had reported consequences of FGM to include pain, excessive bleeding, swollen genital tissue, urine retention, infection, prolonged labour and death as a result of shock due to severe pain or huge loss of blood 45, 46, 47.

Few participants reported difficulty during delivery as one of the consequences of FGM. Prolonged labour, obstetric lacerations, severe heamorrahge, difficult deliveries (delayed second stage of labour) are consequences of FGM 48, resulting from the constriction of the birth canal caused by the narrowed birth canal and FGM scar. This may lead to the death of both the mother and baby if emergency obstetric care is not given. Very few participants admitted knowing association between FGM and woman's sexual desire. The clitoris, however, is one of the erogenous zones in females that play a vital function in the sexual activities of women. When cut off, a woman could find it difficult to enjoy sex 49. Female genital mutilation has mental health implications. Adelufosi et al. 50 asserted that FGM affect the victims mentally and they exhibit some psychological symptoms like depression, anxiety disorders, fear, nightmares, anger, shame, and feeling of helplessness. We found out generally, that our participants do not have good knowledge of health implications of FGM. This could be the main reason the practice is still going on in the communities.

The findings of this study, therefore, brings to the front lines why FGM is still practiced in most rural communities. Knowledge is power and as such determines to a great extent what one does. Programmes that could address the poor knowledge of health implications of FGM down to the rural communities are needed, engaging the traditional leaders and the circumcisers. There is also implication for law enforcement. As earlier stated, Nigeria has a law against FGM, but the problem remains its enforcement. There is a sensitization campaign in the study area, but its major target is not education on health implications of FGM and focus has been mainly on women, when it is more important to educate all adults especially community law makers. Ebonyi State has recently launched a law prohibiting FGM 27. This law instills fears in the community members. During our study, we noticed that because of the law and the sensitization campaign, community members were not comfortable to discuss FGM. Though, these have reduced the prevalence of FGM, they do not however, mean absence of the practice. FGM is done secretly to avert the wrath of the law.

## Conclusion

FGM is still in practice in rural communities in Ebonyi State which maybe because of poor knowledge of health implications of FGM. The type most commonly practiced is clitoridectomy.

## Recommendations

Based on our findings, we recommend the following:

1. Community-based sexuality education programme should be developed and implemented by national and international health related organizations and NGOs. This will help equip the community members with basic knowledge of health consequences of FGM, skills to refuse FGM and strategies to stop the practice.
2. Traditional rulers and law makers should be adequately engaged through advocacy, seminars and workshops to get them abreast with the urgent need to stop FGM.
3. The federal government should enforce the law against FGM in rural communities.

## Limitation of the study

Some of the respondents especially those who were still engaged with FGM were reluctant to divulge information to the interviewers for fear of litigation. Despite all assurances given them that the interview was for research purpose only and that they will not in any way be associated with their responses, some, especially the males still could not openly talk about their practices. This study cannot be generalized to the entire State because participant were not randomly selected. Moreover, the use of interpreters may also have some impacts on the data.

## Figures and Tables

**Table 2:** Major questions for the interviews

Area of Interest	Core questions
Practice	1. What type of female circumcision do you practice in your community? 2. When is female circumcision done in your community? 3. Who performs the female circumcision? 4. What instrument(s) do they use? 5. How are the instruments prepared for use before the circumcision? 6. How is the wound managed? 7. How does your community reward girls that underwent female circumcision? 8. What are the punishments or sanctions given to someone that refused to undergo female circumcision in your community?
Knowledge of Health implications	9. What immediate effect do you notice on the body or site of circumcision? 10. What other later effects of female circumcision do you know, for example during delivery? 11. How can female circumcision affect marital relationship? 12. How can female circumcision affect sexual satisfaction? 13. How do circumcised females feel in your community?
